# SapC-DOPS – a Phosphatidylserine-targeted Nanovesicle for selective Cancer therapy

**DOI:** 10.1186/s12964-019-0476-6

**Published:** 2020-01-09

**Authors:** Kombo F. N’Guessan, Priyankaben H. Patel, Xiaoyang Qi

**Affiliations:** 1 Division of Hematology/Oncology, Department of Internal Medicine, University of Cincinnati College of Medicine, Cincinnati, OH USA; 2 Department of Pathology and Laboratory Medicine, University of Cincinnati College of Medicine, Cincinnati, OH USA; 3 Department of Biomedical Sciences, University of Cincinnati, Cincinnati, OH USA; 4 Division of Human Genetics, Department of Pediatrics, University of Cincinnati College of Medicine and Cincinnati Children’s Hospital and Medical Center, Cincinnati, OH USA; 5 Department of Biomedical Engineering, College of Engineering and Applied Science, University of Cincinnati, Cincinnati, OH USA

**Keywords:** Phosphatidylserine, SapC-DOPS, Flippase, Pancreatic cancer, Lung cancer, Brain tumor

## Abstract

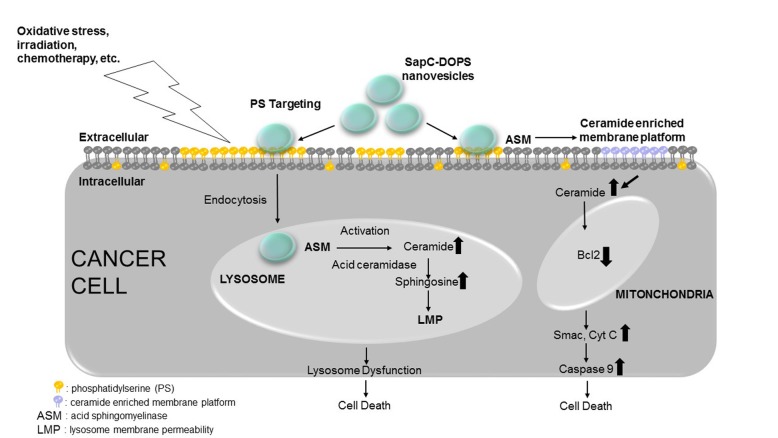

## Background

Phosphatidylserine (PS) is an anionic phospholipid, important for the functioning and integrity of the eukaryotic cellular membrane [1]. PS is normally distributed asymmetrically in the plasma membrane where it is predominantly found in the inner leaflet of the membrane bilayer. This occurs, in part, through the action of flippase complexes, which selectively translocate PS inwards [1–3]. However, in cancer cells, PS is often expressed at high levels on the outer leaflet of the plasma membrane [4–7]. Decreased flippase activity associated with increased influx of Ca^2+^ into cells and oxidative stress, chemotherapy and radiotherapy have all been shown to increase PS expression in the surface of cancer cells [6, 8–10]. The overexpression of PS on the surface of cancer cells has presented an opportunity for selective therapeutic targeting of cancer cells without effecting healthy cells with low surface PS [2, 11]. PS can be used for identification and killing of cancer cells [2, 7, 12–17]. Strategies for achieving this therapeutic effect have included the use of PS-targeting antibodies that block PS-mediated immunosuppression by binding to PS on tumor cells and vasculature; annexins which inhibit tumor angiogenesis by binding to PS on tumor cells; and PS-targeting synthetic peptides which enhance membrane poration through binding to PS leading to increased cell death [18–21]. The focus of this review will be PS-targeting nanovesicles which have emerged as a strategy for selective targeting of high surface PS cancer cells [17]. These nanovesicles target cancer cells based on their binding affinities to PS, allowing them to deliver therapeutic drugs via binding to PS to induce cancer cell death while leaving healthy cells unaffected [2, 11, 17]. Specifically, our lab has focused on the development of SapC-DOPS, a PS-targeting nanovesicle comprised of saposin C (SapC; a lysosomal protein) and dioleylphosphatidylserine (DOPS) [2, 10, 12, 15, 17, 22, 23]. The unique appeal of SapC-DOPS as a cancer therapy include its consistent selective targeting and killing of cancer cells while being tolerated in healthy cells – this phenomenon has been reflected in results of phase I clinical trials where SapC-DOPS showed a strong safety profile [24, 25]. In addition, SapC-DOPS [1] utilizes multiple mechanisms to induce cancer cell death including caspase 9 cleavage and lysosomal membrane permeability [2] is capable of crossing the blood-brain tumor barrier and [3] enhances the effects of existing therapies [9, 10, 23, 26]. Therapeutic assessment of SapC-DOPS and other PS-targeting nanovesicles by other researchers indicate that, as a class, they are a promising therapeutic option for the treatment of several types of cancers.

## SapC-DOPS

SapC-DOPS is a nanovesicle derived from sphingolipid activator protein C (SapC) and dioleylphosphatidylserine (DOPS) [2, 17, 27–31]. At low pH ranges, SapC and DOPS spontaneously form nanovesicles with a mean diameter of approximately 200 nm. Saposin C is one of four small glycoproteins derived from the cleavage of the saposin precursor, prosaposin. It is a heat-stable, protease-resistant, non-enzymatic activator of lysosomal enzymes [2, 17, 27–31].

**Fig. 1:**
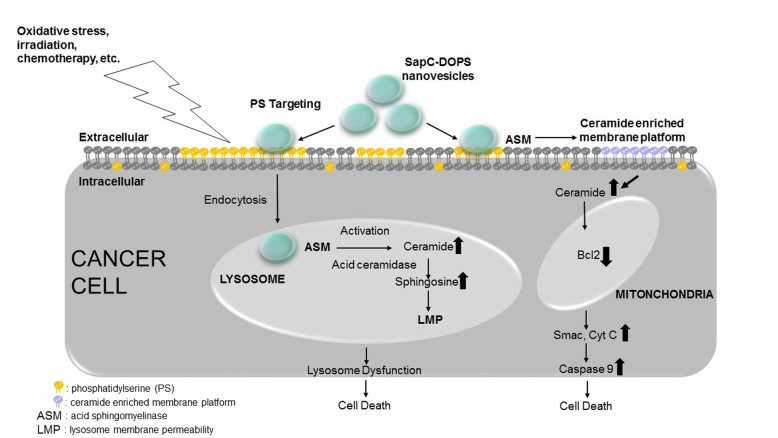
Schematic figure depicting the mechanism of action of SapC-DOPS. Externalization of PS to the surface of cancer cells occurs through several pathways including oxidative stress, irradiation and chemotherapy. SapC-DOPS targets PS-rich membrane surfaces and induces cancer cell death by activating acid sphingomyelinase and increasing ceramide levels in the cell. This increase in ceramide levels induces cell death by (1) inducing lysosome membrane permeability and (2) inducing cytosolic release of Smac and Cyt C leading to caspase 9 cleavage and apoptosis

Binding of SapC-DOPS to cancer cells is dependent on PS expression on the surface of cells [2, 7, 10, 14–16, 22, 32–34]. Following PS binding, SapC undergoes conformational changes resulting in the reorientation of its functional helical domains. SapC requires direct binding and interaction with PS to exert its enzyme-activating activity [2, 17]. Importantly, the higher the PS expression on the surface of a cell, the more effectively SapC-DOPS binds to the cell and triggers the ceramide cascade, ultimately resulting in apoptosis (Fig. 1) [15, 26]. Binding of SapC to PS is favored at acidic pH. Similarly, studies of SapC-DOPS in lung cancer cells have revealed that SapC–DOPS binding to cancer cells is more pronounced at low pH [15]. As the tumor microenvironment is acidic [35, 36], SapC-DOPS will especially target the tumor’s surface PS while leaving normal tissues (with neutral pH) alone. The activation of acid sphingomyelinase by SapC leads to intracellular production and accumulation of ceramide, and subsequent apoptosis [37]. Studies in neuroblastoma reveal that SapC-DOPS-induced apoptosis involves cytosolic release of second mitochondria-derived activator of caspases (Smac) and cytochrome c, as well as mitochondrial translocation and polymerization of Bax (Fig. 1) [26]. Studies of saposin C membrane fusion revealed that although saposin C-induced fusion occurred with a mixture of anionic saturated and unsaturated acyl chains, the fusion process was much slower than that with synthetic unsaturated DOPS, thus DOPS enhances saposin C fusion, especially at acidic pH [30]. Importantly, when saposin C or DOPS were used individually to treat pancreatic cancer cells, apoptosis was not induced. These results suggest that both Saposin C and DOPS are required for optimal cytotoxic effects of SapC-DOPS [15].

## Theraeutic studies of SapC-DOPS in cancer cells

SapC-DOPS has been shown to effectively bind to, enter and induce cell death in several cancer cell types [7, 9, 10, 12–15, 17, 22, 23, 38]. In vitro, the cytotoxic effect of SapC-DOPS correlated with surface PS expression on cells. Our studies have demonstrated that SapC-DOPS selectively induces apoptotic cell death in malignant pancreatic cells, whereas untransformed pancreatic ductal epithelial cells remain unaffected [13–15, 38]. Furthermore, animals with xenograft tumors treated with SapC-DOPS showed clear survival benefits and reduced tumor size compare to untreated mice. Use of a double-tracking method in live mice showed that the nanovesicles specifically targeted and accumulated in the orthotopically-implanted, bioluminescent pancreatic tumors [13, 15].

In in vivo glioblastoma (GBM) tumors, SapC-DOPS showcased its ability to cross the blood–brain tumor barrier (BBTB) as well as target tumor cells, in vitro [7, 10, 16, 32–34]. Tumor targeting by SapC-DOPS in vivo was inhibited by blocking PS exposed on cells with lactadherin, a PS binding protein [10]. SapC-DOPS exerts strong anti-angiogenic activity both in vitro and in vivo and hypoxic cells are sensitized to SapC-DOPS–mediated killing [10]. SapC-DOPS nanovesicles demonstrated direct cytotoxic activity against metastatic breast cancer cells in vitro and also selectively targeted brain metastases-forming cancer cells both in vitro, in co-cultures with human astrocytes, and in vivo, in mouse models of brain metastases derived from human breast or lung cancer cells [7].

Similar effects were observed in skin, lung and breast cancer cells. Using a cell viability assay (MTT), the cytotoxic effect of SapC-DOPS was tested in three skin tumor cell lines (squamous cell carcinoma, SK-MEL-28, and MeWo) and compared to two normal non-tumorigenic skin cells lines, normal immortalized keratinocyte (NIK) and human fibroblast cell (HFC) [12]. The study revealed that the nanovesicles selectively killed the skin cancer cells whereas untransformed skin cancer cells were unaffected. These results were confirmed in vivo using subcutaneous skin tumor xenografts. We also showed that SapC-DOPS specifically targets human lung cancer xenografts, and that systemic therapy with SapC–DOPS induces tumor death and significantly inhibits tumor growth [22].

The effect of SapC-DOPS has also been assessed in the pediatric cancers using neuroblastoma and peripheral neuroblastic tumors [17, 26]. SapC-DOPS effectively targeted and inhibited the growth of neuroblastoma and pNTS in vitro. Furthermore, xenograft mice were used to demonstrate in vivo therapeutic efficacy. In these mice, SapC-DOPS exhibited specific targeting of neuroblastoma tumors and induced apoptotic cell death [17, 26].

## SapC-DOPS combination treatments

Studies investigating the effects of irradiation on SapC-DOPS cytotoxicity have shown that fractionated radiation enhances the effect of SapC-DOPS in some cancer cell lines [23]. In vitro irradiation of cancer cell lines increased median surface PS expression of surviving cells [9]. The observed effects of increased surface PS and sensitization to SapC-DOPS was most pronounced in cell lines with lower initial surface PS expression. The observed effects were also seen in vivo where radiation increased the surface PS of tumor cells in subcutaneous xenografts in nude mice. The study revealed an inverse relationship between surface PS expression in cancer cells and sensitivity to radiation-induced cell death. Furthermore, serial irradiation which increased overall surface PS expression in surviving cells, increased resistance to radiation and chemotherapeutic drugs. These findings suggest a possible surface PS-based mechanism for radio- or chemo-therapeutic resistance. In addition to irradiation, SapC-DOPS has displayed strong synergistic interactions with the apoptosis-inducing agent, temozolomide (TMZ) in GBM cells although the mechanism behind the synergistic effect has not been fully elucidated [23].

## SapC-DOPS clinical trial

A phase I clinical trial for SapC-DOPS (BXQ-350) was initiated in 2016 for patients with advanced solid tumors and recurrent high-grade gliomas. Phase 1a and 1b studies revealed an impressive safety profile and some efficacy even though treatment was started at very late stages of the disease [24, 25]. Thus far, BXQ-350 has had no serious related adverse events in patients [25].

## Current challenges and future directions

Currently, the majority of studies of PS in the tumor microenvironment are in the context of apoptosis, phagocytosis and immune cell function [4, 6, 39, 40]. However, numerous studies have suggested a potential therapeutic use of PS-targeted nanovesicles such as SapC-DOPS in combination with current cancer treatments for targeting non-apoptotic cancer cells with elevated surface PS expression [9, 23]. As previously stated, cancer cells vary in their expression of surface PS so identification of treatments that elevate surface PS expression in low surface PS cancer cells, such as irradiation, would facilitate targeting of these cells using PS-targeted nanovesicles [9]. PS externalization is generally regulated by increases in intracellular calcium, which inhibits the activity of flippases [8, 41], however the exact mechanism leading to non-apoptotic PS externalization in cancer cells remains unknown. Studies suggest that the mechanisms leading to PS externalization in non-apoptotic cancer cells involve oxidative stress and reduced flippase activity [4, 8, 42]. Oxidative stress in the tumor microenvironment is thought to promote PS externalization to the surface of cancer cells leading to the development of tumor immunity by recognizing and antagonizing activated human blood monocytes [4, 43, 44]. Identifying the mechanism through with oxidative stress leads to non-apoptotic PS externalization in cancer cells will increase our ability to target all cancer cells with SapC-DOPS and other PS-targeting nanovesicles.

In summary, SapC-DOPS has shown the ability to cross the BBTB, allowing for selective killing of brain tumor cells expressing elevated surface PS [7]. However, the mechanism by which this occurs is only partially understood. Understanding the mechanisms involved in facilitating this process will allow for further investigations combining SapC-DOPS with other brain tumor treatments such as previously described with TMZ [23]. While most in vivo studies of SapC-DOPS have been in primary tumor models [9, 10, 15, 22], SapC-DOPS has been shown to be effective against brain metastases-forming cancer cells both in vitro and in vivo [7]. Greater assessment of SapC-DOPS in a metastatic setting would be informative in determining the extent of its effectivity on advanced metastatic cancer disease.

## Conclusions

SapC-DOPS nanovesicles have been shown to successfully target several cancer types in vitro and in preclinical animal models [7, 9, 10, 12, 15, 17]. The nanovesicles are selectively cytotoxic for cancer cells expressing high levels of surface PS [9, 10, 15]. Furthermore, SapC-DOPS nanovesicles are capable of crossing the BBTB [10]. Phase I clinical trials for SapC-DOPS in patients with advanced solid tumors and recurrent high-grade gliomas have revealed an encouraging safety profile [24, 25]. In conclusion, SapC-DOPS is a promising and selective PS-targeting treatment option for several cancers types, worthy of further investigation and clinical development.

## Data Availability

Not applicable.

## Abbreviations

DOPS: Dioleylphosphatidylserine
HFC: Human fibroblast cell
NIK: Normal immortalized keratinocyte
PS: Phosphatidylserine
SapC: Saposin C
TMZ: Temozolomide
